# Wnt Signaling Alteration in the Spinal Cord of Amyotrophic Lateral Sclerosis Transgenic Mice: Special Focus on Frizzled-5 Cellular Expression Pattern

**DOI:** 10.1371/journal.pone.0155867

**Published:** 2016-05-18

**Authors:** Carlos González-Fernández, Renzo Mancuso, Jaume del Valle, Xavier Navarro, Francisco Javier Rodríguez

**Affiliations:** 1 Molecular Neurology Laboratory, Hospital Nacional de Parapléjicos (HNP), Toledo (Spain); 2 Institute of Neurosciences and Department of Cell Biology, Physiology and Immunology, Universitat Autonoma de Barcelona, and Centro de Investigación Biomédica en Red sobre Enfermedades Neurodegenerativas (CIBERNED), Bellaterra, Spain; Institute of Health Science, CHINA

## Abstract

**Background:**

Amyotrophic lateral sclerosis is a chronic neurodegenerative disease characterized by progressive paralysis due to degeneration of motor neurons by unknown causes. Recent evidence shows that Wnt signaling is involved in neurodegenerative processes, including Amyotrophic Lateral Sclerosis. However, to date, little is known regarding the expression of Wnt signaling components in this fatal condition. In the present study we used transgenic SOD1^G93A^ mice to evaluate the expression of several Wnt signaling components, with special focus on Frizzled-5 cellular expression alteration along disease progression.

**Findings:**

Based on previous studies demonstrating the expression of Wnts and their transcriptional regulation during Amyotrophic lateral sclerosis development, we have analyzed the mRNA expression of several Wnt signaling components in the spinal cord of SOD1^G93A^ transgenic mice at different stages of the disease by using real time quantitative PCR analysis. Strikingly, one of the molecules that seemed not to be altered at mRNA level, Frizzled-5, showed a clear up-regulation at late stages in neurons, as evidenced by immunofluorescence assays. Moreover, increased Frizzled-5 appears to correlate with a decrease in NeuN signal in these cells, suggesting a correlation between neuronal affectation and the increased expression of this receptor.

**Conclusions:**

Our data suggest the involvement of Wnt signaling pathways in the pathophysiology of Amyotrophic Lateral Sclerosis and, more specifically, the implication of Frizzled-5 receptor in the response of neuronal cells against neurodegeneration. Nevertheless, further experimental studies are needed to shed light on the specific role of Frizzled-5 and the emerging but increasing Wnt family of proteins research field as a potential target for this neuropathology.

## Introduction

Amyotrophic lateral sclerosis (ALS) is the most common and fatal form of motor neuron (MN) degenerative disease, with an annual incidence of 1–3 cases per 100,000 inhabitants [1]. The development of the pathology is characterized by progressive MN degeneration in the brain and spinal cord, leading to a deterioration of the neuromuscular function and as result weakness, atrophy, paralysis of skeletal muscles and eventually death from respiratory failure within 3 to 5 years from diagnosis [2–6]. The etiology of the disease is unknown in most ALS cases, which are described as sporadic (SALS), whereas a 10–20% of ALS cases are classified as familial (FALS) [1–5]. The first gene to be identified as being mutated in ALS was the cytosolic copper/zinc superoxide dismutase (Cu/Zn SOD, SOD1) [7, 8] and to date, over 150 different mutations in SOD1 have been discovered (http://alsod.iop.kcl.ac.uk/). Interestingly, both SALS and FALS are suggested to have common pathological hallmarks [4, 9]. The SOD1^G93A^ transgenic mice is a valuable tool for ALS research, characterized by over-expression of the mutant human SOD1 and the development of an age-dependent degeneration of MNs, very similar to human ALS in terms of clinical and pathological features, including leading to progressive paralysis and death [10]. MN death seems to be driven by a convergence of damaging mechanisms, including glutamate excitotoxicity, oxidative stress, mitochondrial dysfunction, endoplasmic reticulum stress, defects in RNA processing, neurofilament accumulation, growth factor abnormalities, astroglia and/or microglia dysfunction, defects in axonal transport, metabolic alterations, accumulation of protein aggregates and immune imbalance [3, 6, 9, 11]. However, the exact cellular and molecular mechanisms responsible for MN degeneration in ALS are not completely understood, and to date, there is no cure for this neuropathology.

The Wnt family of proteins plays key roles during central nervous system (CNS) development and adult tissue homeostasis by regulating different cellular processes, such as migration, proliferation, differentiation, polarisation, axonal guidance, cell-cell adhesion and synapse physiology [12–16]. Furthermore, Wnt signaling has been involved in several neuropathologies during adulthood, including Alzheimer [17, 18], Parkinson [19] and Huntington [20] diseases, spinal cord injury (SCI) [21–26] and ALS [27–33]. Briefly, Wnt proteins can modulate, at least, three different signaling pathways. On one hand, in the canonical/β-catenin pathway, Wnt ligands interact with one of the 10 Frizzled (Fz) receptors and a low-density Lipoprotein Receptor-related Protein 5/6 (LRP5/6) co-receptor, leading to active β-catenin translocation to the nucleus and gene expression induction by the interaction with T-cell Factor/Lymphoid Enhancer Factor (TCF/LEF) family of DNA-binding proteins [34–38]. On the other hand, the non-canonical Wnt/Ca2+ and PCP pathways are activated by Fz receptors without LRP involvement, or by different non-conventional receptors, such as Ryk and Receptor Tyrosine Kinase-Like Orphan Receptor (ROR-1/2) [37, 39–41]. Moreover, there are different regulatory mechanisms for the Wnt-mediated signaling, including different extracellular antagonists, such as secreted Frizzled-Related Proteins (sFRPs), Dickkopf (Dkk) or Wnt inhibitory factor 1 (Wif-1) [42, 43].

Interestingly, recent results demonstrate the expression of different Wnt signaling components in the spinal cord of ALS transgenic mice, with variations in several gene expression levels during the progression of the disease [27–33]. To gain further understanding of involvement of Wnt mediated pathways in the pathogenesis of ALS, we analyzed the expression of several Wnt signaling components in the spinal cord of ALS transgenic mice at different stages by quantitative RT-PCR (RT-qPCR) and immunohistochemistry. Then, we determined the cellular expression pattern of Fz5 and its cellular protein expression changes at different stages of the pathology. Further experimental studies must be performed to determine the molecular mechanisms underlying the changes found and their role in the pathogenesis of ALS, which may lead to new strategies for treating neurodegenerative diseases through modulation of Wnt signaling pathways.

## Materials and Methods

### Animals

Experiments were performed in transgenic mice carrying the mutation G93A in SOD1 gene and in nontransgenic Wild-Type (WT) littermates as controls. Transgenic mice with the G93A human SOD1 mutation (B6SJL-Tg[SOD1-G93A]1Gur) were obtained from the Jackson Laboratory (Bar Harbor, ME), breaded at the animal facilities of the Universidad de Zaragoza (Spain) and maintained at the Universitat Autònoma de Barcelona (Spain). Hemizygote B6SJL SOD1^G93A^ males were obtained by crossing with B6SJL females from the CBATEG (Barcelona, Spain). The offspring was identified by PCR amplification of DNA extracted from the tail tissue. Mice were kept in standard conditions of temperature (22 ± 2°C) and a 12:12 light:dark cycle with access to food and water *ad libitum*. All experimental procedures were approved by the Ethics Committee of the Universitat Autònoma de Barcelona. Studies were performed in 16 weeks old WT and 8, 12 and 16 weeks old SOD1^G93A^ mice (n = 6 each), representing pre-symptomatic, clinical symptoms onset and end stage of the disease, respectively [44–48].

### RNA isolation and RT-qPCR analysis

At each of the time points chosen for study, animals were terminally anesthetized with pentobarbital and perfused intracardially with heparinized saline to remove blood from the tissue. Lumbar spinal cords were harvested and the anterior horns were gently dissected under a microscope. Total mRNA was isolated by using the RNeasy Mini Kit (Qiagen). Complementary DNA (cDNA) synthesis from DNase-treated RNA (0.5 μg) and the relative quantifications were performed as previously described [23] using 50 ng of cDNA and specific primers validated previously [26]. β-actin (forward 5’-AAGTCCCTCACCCTCCCAAAAG-3’, reverse 5’- AAGCAATGCTGTCACCTTCCC-3’. GenBank accession number NM_007393.3) was quantified in a separate well as real-time reporter.

### Immunohistochemistry

Similarly, three WT, and 8w, 12w and 16w old SOD1^G93A^ mice were intracardially perfused with heparinized saline solution followed by 4% paraformaldehyde. The lumbar spinal cords were harvested and then post-fixed for 4h in 4% paraformaldehyde, cryoprotected by immersion in 30% sucrose for 48h, embedded in OCT frozen medium (Sakura). Ten series of 40μm transverse sections were cut using a cryotome (Leica) and collected serially in Olmos solution.

The cellular protein expression and localization of Fz1, Fz4 and Fz5 in both WT and transgenic mice spinal cord was performed by immunofluorescence assays following the same protocol detailed in a previous report [23]. The following primary antibodies produced in rabbit and obtained from Abcam were used: anti-Fz1 (1:50; ab71342), anti-Fz4 (1:5000; ab83042) and anti-Fz5 (1:100; ab75234), followed by the corresponding Dylight594 (1:500; Abcam, ab96897) anti-rabbit. All sections used were processed at the same time and following the same experimental protocol.

To determine the cellular Fz5 expression pattern, double immunofluorescence was performed by combining Fz5 staining with specific astrocytic (glial fibrillary acidic protein; GFAP), neuronal (neuronal nuclei; NeuN) and MN (choline acetyltransferase; ChAT) markers, and also with the mitochondrial SOD2 staining in MNs. The sections were processed as detailed above for Fz5 detection, and the following primary antibodies were used for the different cell types: mouse anti-GFAP (1:1000; Sigma, G3893), mouse anti-NeuN (1:250; Millipore, MAB377), mouse anti-SOD2 (1:100; Abcam, ab110300) and goat anti-ChAT (1:100; Millipore, AB144P). Subsequently, the corresponding Dylight488-linked anti-mouse (1:500; Abcam, ab96879) or Alexa488-linked anti-goat (1:1000; Life technologies, A11055) secondary antibody was used.

Finally, sections were analyzed using both the BX61 Motorized Research Microscope (Olympus) and a Leica TCS SP5 resonant scanner confocal microscope (Leica Microsystems). For single immunohistochemistry experiments, the sections processed without the primary antibody were used as controls. For double immunohistochemistry, in order to confirm lack of cross reactivity, sections were processed without the second primary antibody and used as controls. No non-specific staining was observed.

### TUNEL Assay

Terminal deoxynucleotidyl transferase-mediated dUTP nick end labeling (TUNEL) assay was performed to identify cells undergoing apoptosis. Using the In Situ Cell Death Detection Kit, TMR red from Roche (no. 12156792910), spinal cord sections were treated according to manufacturer’s instructions. For dual immunofluorescence, spinal cord sections were then exposed to Fz5 antibody as described above. As negative control, we omitted the TdT enzyme in the labeling reaction mixture, and, as a positive control, we pretreated sections with DNase I and induced TUNEL positivity in all cells.

### Statistical analysis

All values are expressed as the mean ± SEM. Statistical comparisons were performed using one-way ANOVA, followed by Tukey’s post-hoc test to determine the individual differences between the means. In all cases, p < 0.05 was considered to be statistically significant. All statistical analyses were performed using GraphPad Prism (version 6.01).

## Results

### The Wnt family mRNA expression is modulated by the progression of the ALS pathology

**Fig 1:**
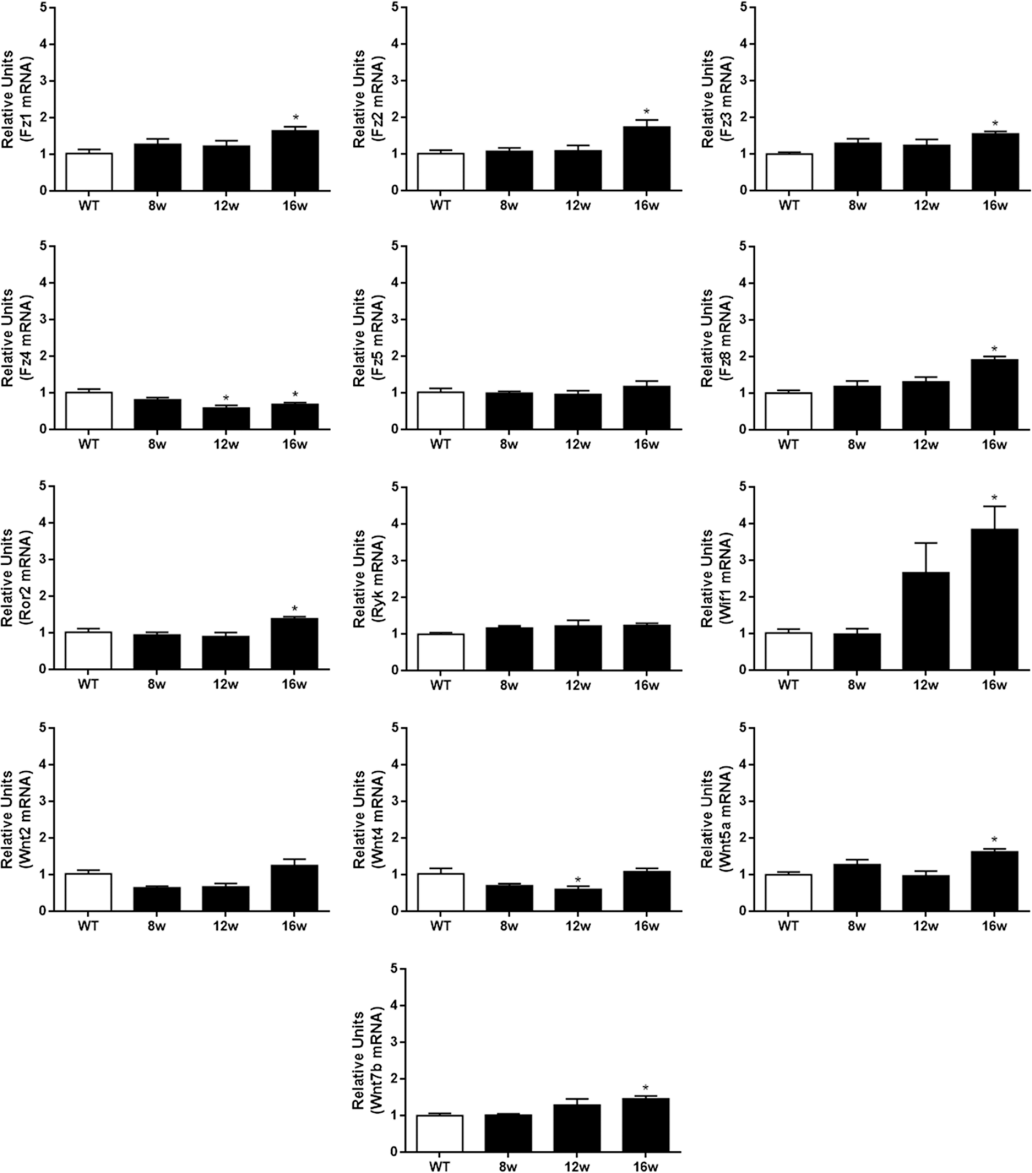
Temporal mRNA expression pattern of different Wnt family members in the spinal cord of both WT and ALS transgenic mice. The temporal mRNA expression pattern of *Fz1*, *Fz2*, *Fz3*, *Fz4*, *Fz5*, *Fz8*, *Ror2*, *Ryk*, *Wif1*, *Wnt2*, *Wnt4*, *Wnt5a* and *Wnt7b* was quantified using real time quantitative PCR using specific primers, both in the WT and ALS transgenic mice spinal cord and at different stages of the disease. Differences were calculated by setting the expression values of the WT samples at 1 and normalising against ribosomal 18S rRNA. In all cases, the data are presented as the mean ± SEM; * p < 0.05 versus WT.

Based on previous findings on Wnt family expression in ALS [27–33], we first assessed the mRNA profile of a set of Wnt family members with reported changes of expression associated to the disease progression. We evaluated the mRNA expression of *Wnt2*, *-4*, *-5a* and *-7b* ligands; *Fz1*, *-2*, *-3*, *-4*, *-5*, *-8*, *Ror2* and *Ryk* receptors; and the *Wif1* secreted inhibitor in both WT and ALS transgenic mice. Between 8w and 12w, most of the molecules analyzed did not show significant changes in their expression levels, and only *Wnt4* and *Fz4* were significantly down-regulated at 12w (Fig 1). In contrast, the majority of significant changes were observed at the later stage of the pathology, at 16w. The mRNA expression of *Wnt5a*, *Wnt7b*, *Fz1*, *Fz2*, *Fz3*, *Fz8*, *Ror2* and *Wif1* was significantly up-regulated when compared to the levels detected in WT mice. Moreover, the mRNA expression of *Fz4* was significantly down-regulated at this time point (Fig 1).

### Protein expression of Fz1, Fz4 and Fz5 receptors and increased immunoreactivity of Fz5 in the spinal cord of ALS transgenic mice

**Fig 2:**
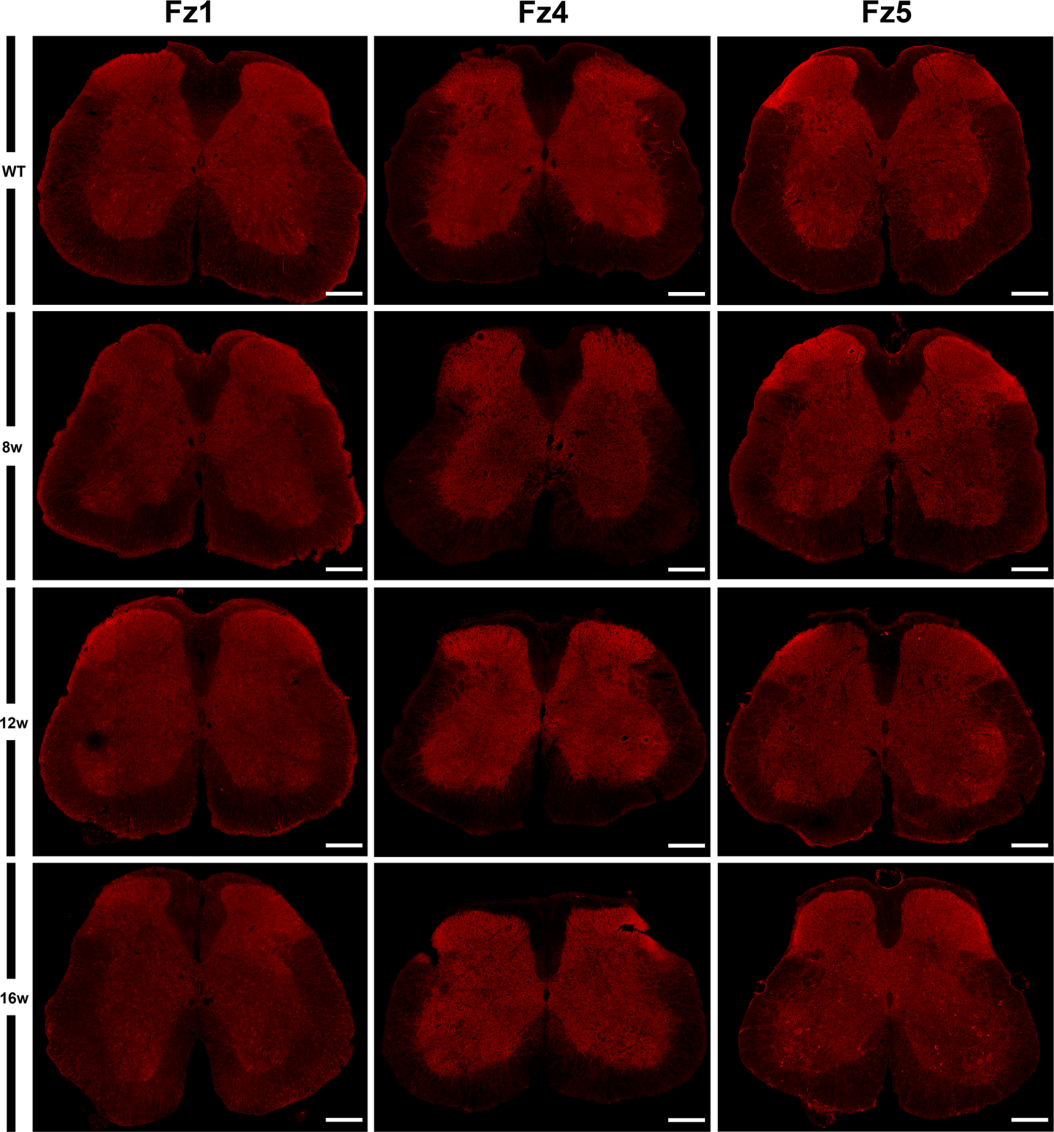
Protein expression of Fz1, Fz4 and Fz5 receptors in the spinal cord of both WT and ALS transgenic mice. This figure shows representative images obtained from the microscopic evaluation of sections processed by simple immunofluorescence to visualize Fz2, Fz4 and Fz5. Fz1 and Fz4 receptors do not undergo changes in their cellular expression pattern during disease progression. Instead, Fz5 immunoreactivity begins to increase at 12w in some cells, and clearly observed at 16w. The analysis was performed in WT and ALS mice spinal cords at 8w, 12w and 16w. Scale bars = 200 μm.

**Fig 3:**
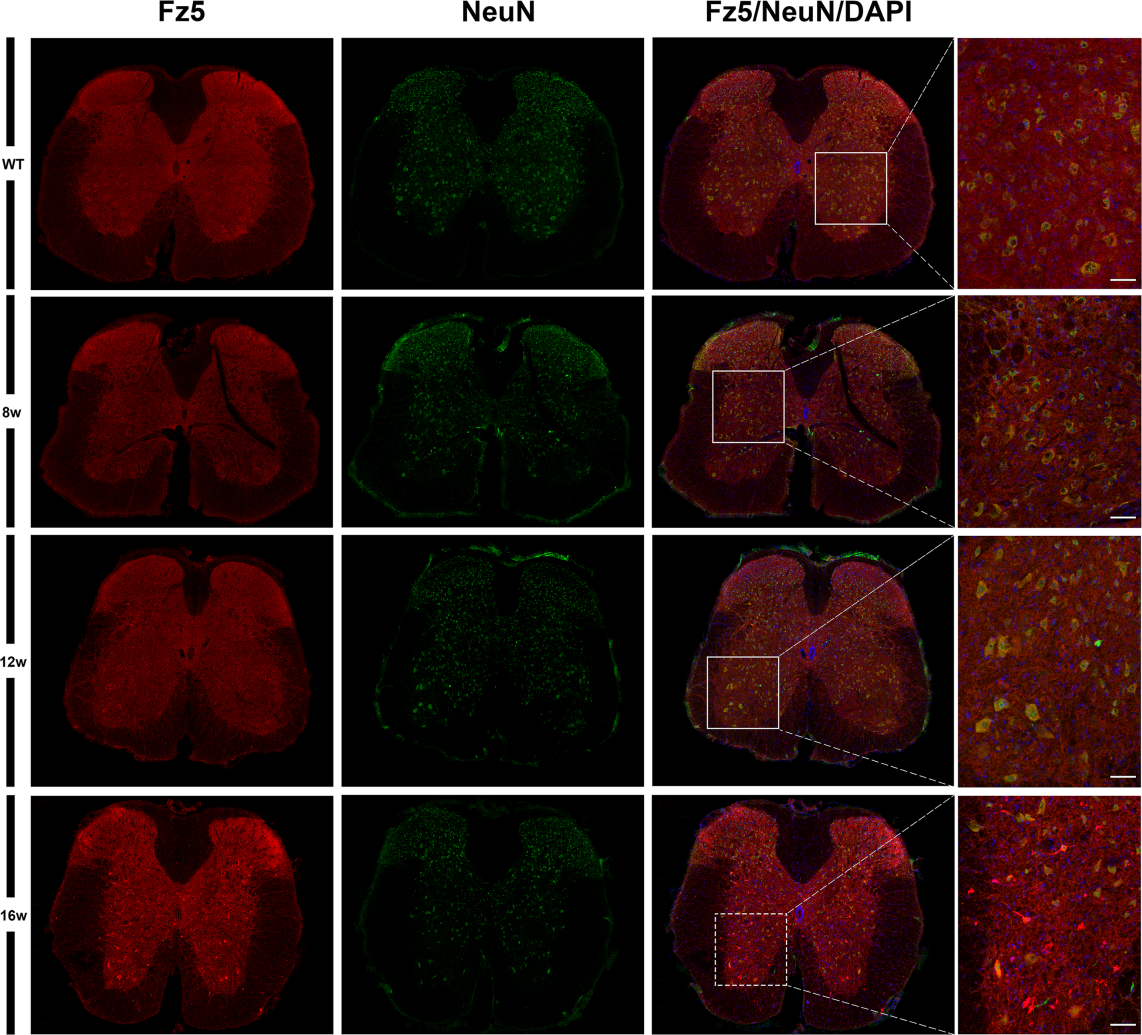
Neuronal location of Fz5 receptor in the spinal cord of both WT and ALS transgenic mice. This figure shows representative images obtained from the microscopic evaluation of sections processed by double immunohistochemistry to visualize Fz5 in neurons (NeuN). Fz5 was expressed in most of neurons at differents spinal cord laminae in WT mice and also at different neuropathological stages of ALS transgenic mice. The analysis was performed in WT and ALS mice spinal cords at 8w, 12w and 16w. The squares in the images showing the entire spinal cord sections represent the areas where the corresponding higher magnification micrographs were obtained. Scale bars = 50 μm.

**Fig 4:**
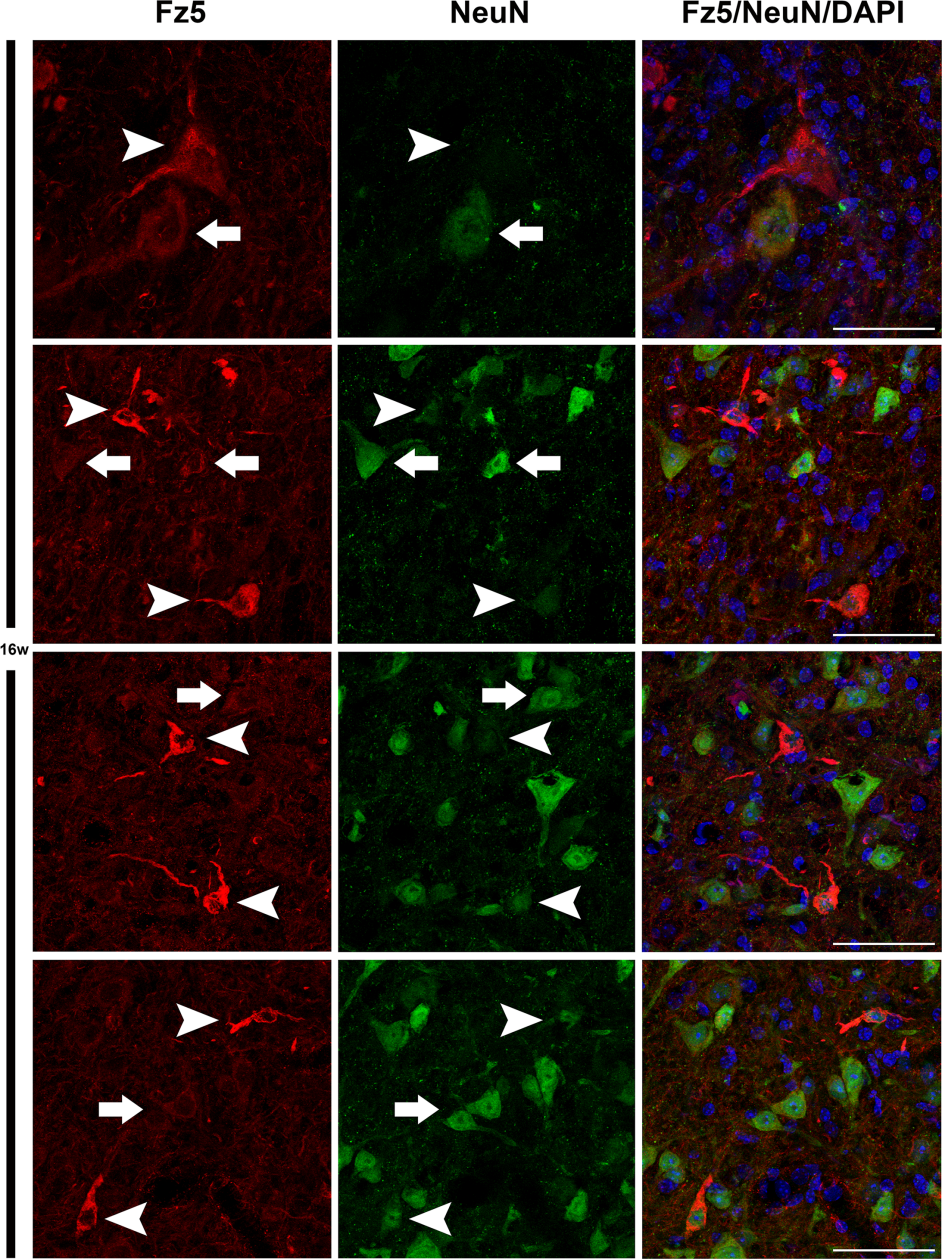
Increased expression of Fz5 receptor matched with a decrease in NeuN immunoreactivity at 16w. This figure shows representative images obtained from the microscopic evaluation of sections processed by double immunohistochemistry to visualize Fz5 in neurons (NeuN). Cells with increased immunoreactivity for Fz5 showed low levels of NeuN staining (arrowheads). However, neurons with a baseline of Fz5 immunoreactivity exhibited normal NeuN expression (arrows). The analysis was performed in 16 ALS mice spinal cords. Scale bars = 50 μm.

**Fig 5:**
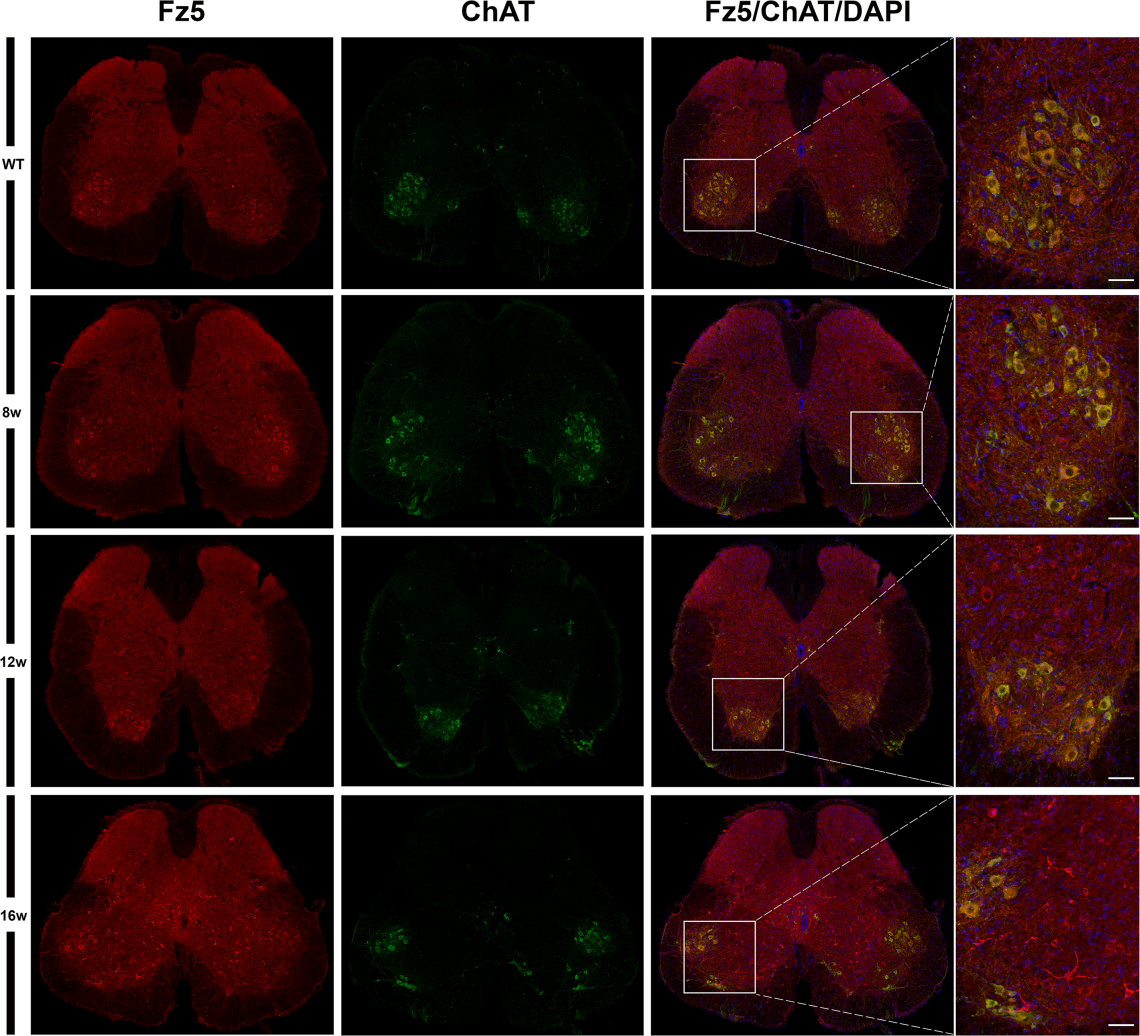
Fz5 receptor was localized in motoneurons. This figure shows representative images obtained from the microscopic evaluation of sections processed by double immunohistochemistry to visualize Fz5 in MNs (Chat). Fz5 receptor was expressed by MNs immunopositive for Chat staining both WT and ALS transgenic mice. No changes were found regarding to any increase in Fz5 immunoreactivity associated with MNs. The analysis was performed in WT and ALS mice spinal cords at 8w, 12w and 16w. The squares in the images showing the entire spinal cord sections represent the areas where the corresponding higher magnification micrographs were obtained. Scale bars = 50 μm.

Next, using single immunohistochemistry, we investigated the protein expression of Fz1, Fz4 and Fz5, as representatives of an increase, a decrease and no change in mRNA expression levels. As previously reported by our group, Fz1 was expressed by neurons and oligodendrocytes while Fz4 was expressed by astrocytes [26] in the spinal cord of WT animals, a cell pattern and distribution that was unaltered in the transgenic mice. By contrast, Fz5 showed changes in its expression pattern during the progression of the disease (Fig 2), so the rest of experiments focused in this receptor. We found an increase in the immunoreactivity of Fz5 receptor in SOD1 mice spinal cord concomitant to disease progression, which seems to specifically affect neurons located in different layers of the spinal gray matter (Fig 3). Interestingly, this increase seemed to match with a decrease in NeuN immunoreactivity in these same cells (Fig 4). Indeed, Fz5 expression in the ventral horn of the spinal gray matter co-localized with the MN marker ChAT (Fig 5) of both WT and SOD1 transgenic mice at the different times analyzed. However, MNs were not among the cells with increased Fz5 immunoreactivity in the spinal cord of ALS transgenic mice.

**Fig 6:**
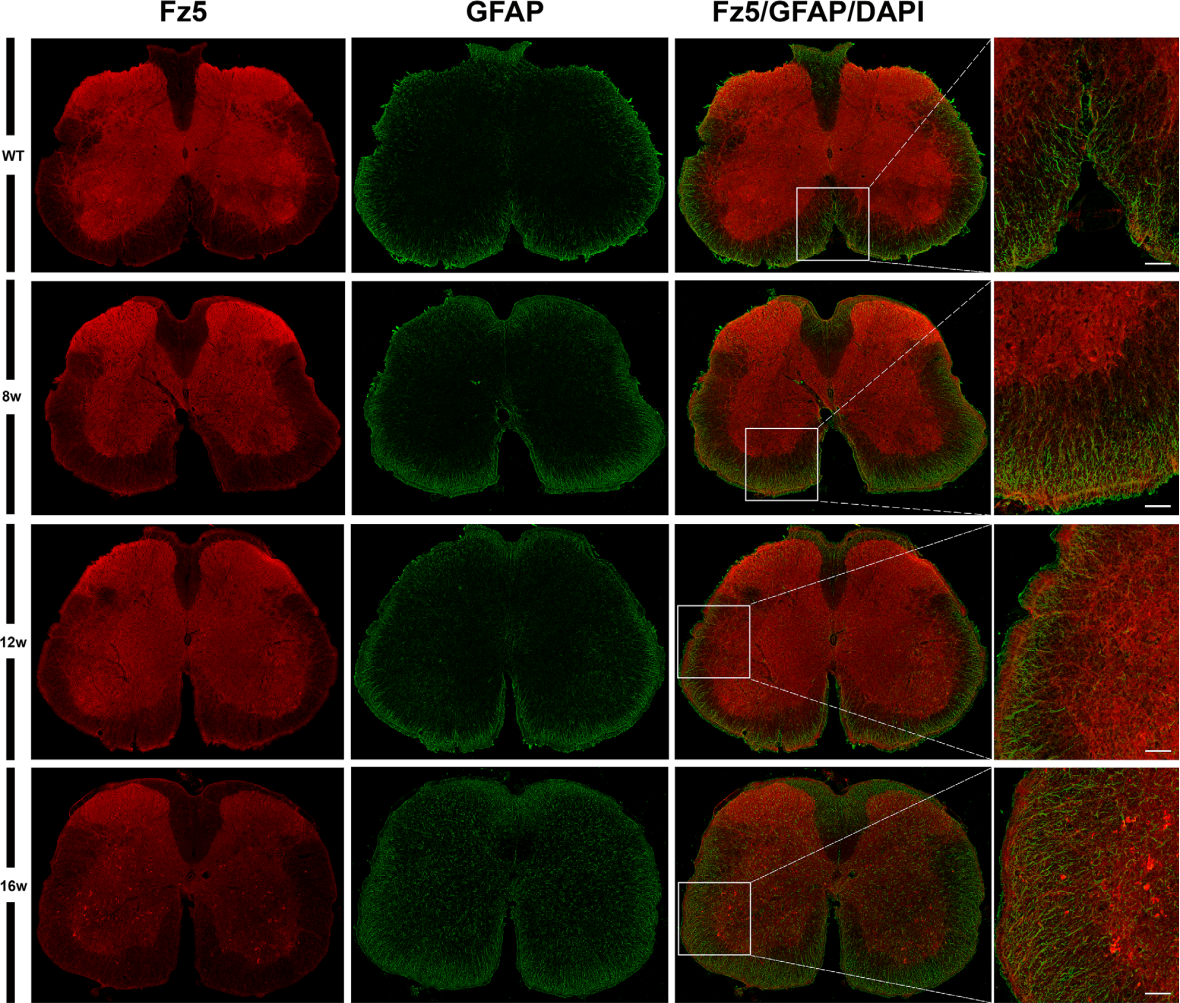
Fz5 receptor was localized in astrocytes. This figure shows representative images obtained from the microscopic evaluation of sections processed by double immunohistochemistry to visualize Fz5 in astrocytes (GFAP). Fz5 receptor was located in astrocytes both WT and ALS transgenic mice, mainly in contacting pial surface regions, but no changes were observed during disease progression. The analysis was performed in WT and ALS mice spinal cords at 8w, 12w and 16w. The squares in the images showing the entire spinal cord sections represent the areas where the corresponding higher magnification micrographs were obtained. Scale bars = 50 μm.

Fz5 was also located in astrocytes of both WT and SOD1 transgenic mice, mainly in the regions in close contact with the pial surface. However, we did not observe variations in protein expression of Fz5 in astrocytes, since immunoreactivity levels of the receptor do not seem to be altered at any of the stages evaluated for this condition, in contrast with the remarkable increase of GFAP expression or glial reactivity observed at advanced stages of the disease (Fig 6).

**Fig 7:**
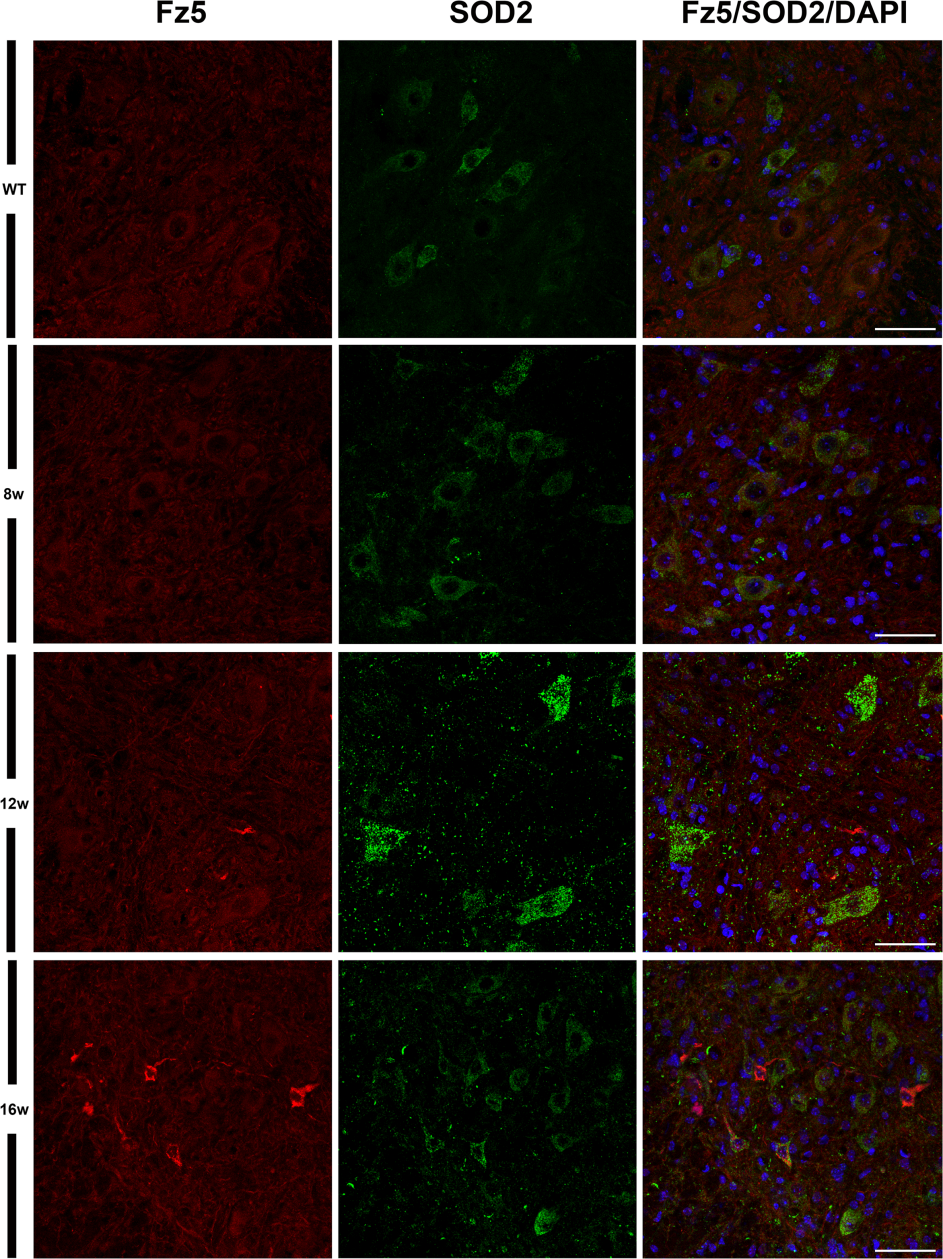
Location of Fz5 receptor and the mitochondrial enzyme SOD2. This figure shows representative images obtained from the microscopic evaluation of sections processed by double immunohistochemistry to visualize Fz5 and mitochondrial enzyme SOD2. We did not observe a correlation between increased immunoreactivity of Fz5 and SOD2 in these neuronal cells, but an increase of SOD2 signal with the progression of the disease was observed. The analysis was performed in WT and ALS mice spinal cords at 8w, 12w and 16w. Scale bars = 50 μm.

Although loss of MNs is the main characteristic of ALS, there is no conclusive evidence that apoptosis is involved [49, 50]. We performed TUNEL staining to look for evidences of nuclear DNA breakdown and identify cells undergoing apoptosis. Nevertheless, no TUNEL-positive neurons were identified in the spinal cords of transgenic mice (data not shown) at the different times analyzed. Furthermore, based on the literature that describes an increase of the SOD2 mitochondrial enzyme associated with neurodegenerative processes [51], we performed double immunohistochemistry assays to evaluate if there was any correlation between increased expression of Fz5 and SOD2 in these same cells. As expected, we found an important increase in the expression of SOD2 in correlation with the progression of the disease, however, we did not observe a clear correlation regarding co-localization with Fz5 (Fig 7).

## Discussion

ALS is the most common form of MN disease characterized by both upper (corticospinal/corticobulbar) and lower (spinal/bulbar) MN degeneration [11]. To date, the only available treatment for ALS is riluzole, an antiglutamatergic agent that blocks the presynaptic release of glutamate, but this treatment only slows down the progression of the disease and gives the patient few months of extended life span [52, 53]. However, the causes of the disease and the mechanisms causing premature death of MN are not clear. Interestingly, recent studies have provided experimental evidences suggesting the involvement of the Wnt family of proteins in the progression of some neuropathologies [17–26], including ALS [27–33]. In this study, we found that several Wnt signaling components were differentially expressed at mRNA level in the spinal cord of SOD1^G93A^ mice at different stages of the disease. In agreement with previous studies in the literature, we have identified an increase in mRNA expression of most of these molecules at late stages. Only *Wnt4* at 12w and *Fz4* at 12–16w showed significant decreases in the expression of their mRNA. Noteworthy, these results do not exactly match a previous study [29], but is important to point out that both time points for the different stages of the disease and the PCR technique (mice specific array for Wnt signaling pathway) differ from ours.

When we investigated the protein expression of Fz1, Fz4 and Fz5 as representatives of the different mRNA expression patterns, we found that Fz1 and Fz4 showed no changes in protein expression during the progression of the disease. Indeed, we have recently described the cellular expression pattern of Fz1 and Fz4 in spinal cord of healthy mice and after spinal cord injury [26], and in that study we neither found differences in the immunoreactivity of these receptors, although there were also variations on their levels of mRNA expression. Interestingly, the expression of Fz1 has been recently associated to neural progenitor cells and immature neurons in adult mice brain [54]. By contrast, Fz5 receptor, whose mRNA expression was not modified throughout symptoms progression, showed a clear alteration in its protein expression at cellular level. We found that Fz5 co-localized with MNs immunopositive for ChAT staining, and also with other neurons expressing the neuronal marker NeuN. Furthermore, from 12w and particularly at 16w, we observed a marked increase in the immunoreactivity of Fz5 in cells that co-express NeuN, and in others that appear to be losing the expression of this neuronal marker. In this regard, there is experimental evidence showing a decrease in NeuN immunoreactivity specifically in neurons that are undergoing a degenerative process [55–57]. Moreover, by using SOD2 immunoreactivity to label mitochondrial swelling [51], we investigated if those neurons showed this sign of damage, but we did not find a correlation in this sense. Neither was found these cells undergoing apoptosis since TUNEL assays failed to identify TUNEL-positive neurons in the spinal cords of transgenic mice at the different times analyzed (data not shown). This observation is in agreement with other studies which also did not found evidence of apoptosis [58–61], despite the existence of conflicting data regarding the activation of apoptotic pathways responsible for MN degeneration in ALS [49, 61, 62]. Therefore, the significance of the clear increase in Fz5 immunoreactivity associated to ALS progression is currently unknown.

The expression of Fz5 has been related with neuron survival and function. In this regard, a recent study described the necessary expression and activity of Fz5 receptor for neuronal survival in the parafascicular nucleus of the thalamus under physiological conditions, suggesting a role of this receptor in neural cell survival [63]. Other studies have described the participation of this receptor in synaptic physiology, being localized at both pre- and post-synaptic sites [64], and also in the establishment of neuronal morphogenesis and polarity in cultured hippocampal neurons [65]. Therefore, the observed increase in Fz5 immunoreactivity during the evolution of the pathology might be related with the survival and/or the synaptic maintenance of neuronal cells involved in the pathophysiology and the progression of the disease. In this regard, it is known that ALS involves degeneration of MNs, as mentioned above, but is also characterized by loss or changes in other types of neurons [66], and it has been described that abnormalities in the activity of interneurons that synapse onto MNs might lead to hyperexcitability and excitotoxicity, contributing to degeneration in ALS [51, 67, 68].

The pathophysiology of ALS also involves non-neuronal cells [11]. Accordingly, we have also investigated if there was any alteration in Fz5 immunoreactivity in astrocytes, since there are evidences in the literature showing the potential toxic role of astrocytes for MNs in ALS [1, 69–71]. We observed a clear up-regulation of GFAP concomitant with the evolution of the disease, but no changes in Fz5 immunoreactivity were detected. The expression of Fz5 seems to be restricted to those astrocytes located in close contact with the pial surface.

In summary, our findings match with other reports describing a differential expression of the Wnt signaling components in the spinal cord of SOD1^G93A^ mice. In particular, this is the first report describing a disturbed Fz5 cellular distribution in good correlation with the progression of ALS, which might be indicative of a pathophysiological role in those neurons with increased levels of expression. Nevertheless, further studies are needed to characterize the individual role of this receptor in the spinal cord physiology and ALS pathophysiology, to eventually lead to the development of novel therapeutic strategies.
